# [Retracted] Knockdown of lncRNA CCEPR suppresses colorectal cancer progression

**DOI:** 10.3892/etm.2026.13309

**Published:** 2026-09-22

**Authors:** Yuxia Duan, Zhixue Fang, Zeya Shi, Ling Zhang

Exp Ther Med 18:3534–3542, 2019; DOI: 10.3892/etm.2019.7942

Following the publication of the above paper, a concerned reader drew to the Editor’s attention that the N-cadherin blots featured in the western blots in Fig. 4C on p. 3540 were remarkably similar to blots that already under consideration for publication in the same journal (*Experimental and Therapeutic Medicine*) in an article written by different authors at a different research institute (albeit the city was the same). In addition, upon analyzing the data in this paper independently in the Editorial Office, it came to light that certain of the invasion assay data featured in Fig. 3D were also strikingly similar to data which appeared in another article written by different authors in the journal *Bioscience Reports*, which has subsequently been retracted.

Owing to the fact that the contentious data described above were already under consideration for publication at the time that this paper was submitted to *Experimental and Therapeutic Medicine*, the Editor has decided that this paper should be retracted from the Journal. After contacting the authors, they accepted the decision to retract the paper. The Editor apologizes to the readership for any inconvenience caused.

